# Ectopic Expression of JcWRKY Transcription Factor Confers Salinity Tolerance via Salicylic Acid Signaling

**DOI:** 10.3389/fpls.2016.01541

**Published:** 2016-10-17

**Authors:** Parinita Agarwal, Mitali Dabi, Komal K. Sapara, Priyanka S. Joshi, Pradeep K. Agarwal

**Affiliations:** ^1^Plant Omics Division, Central Salt and Marine Chemicals Research Institute (CSIR) – Council of Scientific and Industrial ResearchBhavnagar, India; ^2^Academy of Scientific and Innovative Research, Central Salt and Marine Chemicals Research Institute –Council of Scientific and Industrial ResearchBhavnagar, India

**Keywords:** *Jatropha*, JcWRKY, phytohormones, ROS homeostasis, salicylic acid, salinity, transcription factor(s), transgenic

## Abstract

Plants, being sessile, have developed intricate signaling network to specifically respond to the diverse environmental stress. The plant-specific WRKY TFs form one of the largest TF family and are involved in diverse plant processes, involving growth, development and stress signaling through auto and cross regulation with different genes and TFs. Here, we report the functional characterization of a salicylic acid -inducible JcWRKY TF. The JcWRKY overexpression confers salinity tolerance in transgenic tobacco, as was evident by increased chlorophyll content and seed germination potential. The transgenic plants showed increased soluble sugar, membrane stability, reduced electrolyte leakage and generation of reactive oxygen species (H_2_O_2_ and $O_{2}^{•-}$) as compared to the wild type. Furthermore, the low SA treatment along with salinity improved the tolerance potential of the transgenics by maintaining ROS homeostasis and high K^+^/Na^+^ ratio. The transcript expression of SA biosynthetic gene *ICS1* and antioxidative enzymes (*CAT* and *SOD*) showed upregulation during stress. Thus, the present study reflects that JcWRKY is working in co-ordination with SA signaling to orchestrate the different biochemical and molecular pathways to maneuvre salt stress tolerance of the transgenic plants.

## Introduction

Plants with their sessile and autotrophic lifestyle are exposed to multiple stresses simultaneously such as drought, light, temperature, insects, and microbes. These factors are the major constraints/challenge to agriculture productivity. The potential of the sessile plants to survive, adapt and reproduce/multiply, while being exposed to the various environmental challenges is a fascinating process involving a complex array of physiological and biochemical mechanisms. The complex network of stress-responsive signal transduction pathways, converge and diverge in co-ordination toward alleviating and providing abiotic and biotic stress tolerance (Fujita et al., 2006). Transcription factors (TFs) are important for providing specificity toward stress responses (Vaahtera and Brosche, 2011) and conferring multiple stress tolerance via inducing and/or repressing the expressions of an array of downstream genes (Agarwal and Jha, 2010; Pardo, 2010; Shukla et al., 2015). The stress-related TFs such as NAC, MYB, DREB, and WRKYs have been isolated from different plant species and overexpressed in homologous and heterologous systems to genetically engineer stress tolerance (Agarwal et al., 2013). The genome-wide analysis of *Arabidopsis* genome show the presence of 1,510–1,581 (20%) TF genes, of them 45% are plant specific (Iida et al., 2005). Accordingly, the rice genome contain 1,611 TFs (Xiong et al., 2005). Interestingly, the genome of single-celled yeast *Saccharomyces cerevisiae* contains only 12% of TFs (Mewes et al., 1997), indicating the expansion of TFs in plants probably due to significant variability and complexity of plant metabolism, as compared with other organisms (Shiu et al., 2005).

The WRKY family of TFs play an intricate role in regulating the stress signaling pathways by autoregulation or may be by cross regulation through interaction with other proteins like MAP kinases, MAP kinase kinases, Calmodulin, histone deacetylases, etc. for carrying out diverse plant functions (Rushton et al., 2010). WRKY proteins have been implicated in the regulation of different developmental processes such as trichome, seed development and leaf senescence (Johnson et al., 2002; Luo et al., 2005) but their major role is studied and identified during abiotic and biotic stresses (Pandey and Somssich, 2009). Although, WRKY TFs are considered to be plant specific, however, their presence have been reported from unicellular algae, slime mold, and gymnosperms. The WRKY TFs have been reported from *Arabidopsis*, rice (74, 102 Baranwal et al., 2016), soybean (197, Schmutz et al., 2010), papaya, poplar, sorghum, *Physcomitrella patens* (66, 104, 68, 38, respectively, Pandey and Somssich, 2009) and *Jatropha* (58, Xiong et al., 2013). The WRKY family is acquires its name from the highly conserved 60 amino acid stretched WRKY domain, consisting of the highly conserved WRKYGQK at N-terminus and a novel metal chelating zinc finger signature at C-terminus. Eulgem et al. (2000) classified the WRKY TFs into three major groups depending on the number of WRKY domains and the pattern of their zinc finger motifs. The group I WRKY’s exhibit two WRKY domains, whereas, the groups II and III WRKY’s contain only a single domain. However, groups I and II have a C_2_H_2_-type zinc finger motif (C–X_4-5_–C–X_22-23_–H–X–H) with only the C-terminal domain participating in DNA binding activity. The group III differs from I and II in its C_2_HC zinc finger motif (C–X_7_–C–X_23_–H–X–C). Most of the WRKY’s possess a basic nuclear localization signal. The WRKY proteins bind specifically to the W-box *cis*-element TTGAC(C/T) found in the promoters of a large number of plant defense-related genes (Eulgem et al., 2000), including WRKY itself.

The WRKY TFs possess, integrate and cross-talk with a number of signal transduction pathways, and are being studied and characterized for providing multiple stress tolerance. The genome wide analysis of WRKY TF from *Jatropha* have shown that 47 genes are regulated by abiotic stress (Xiong et al., 2013). In our earlier work (Agarwal et al., 2014) we have isolated WRKY TF from *Jatropha curcas* (*JcWRKY*) closely related to group II, an important biofuel crop growing in the wastelands of India. The *JcWRKY* transcript showed upregulation in response to multiple stresses, furthermore, the JcWRKY recombinant protein showed binding to W-box element of pathogenesis related-1 (PR-1), and iso1 (encoding isoamylase1) promoters. In present study, we show that overexpression of *JcWRKY* in tobacco leads to enhanced salinity stress tolerance, and resistance toward salinity is mediated via SA-induced ROS homeostasis in transgenic plants.

## Materials and Methods

### Construction of Plant Transformation Vector and Tobacco Transformation

**FIGURE 1:**
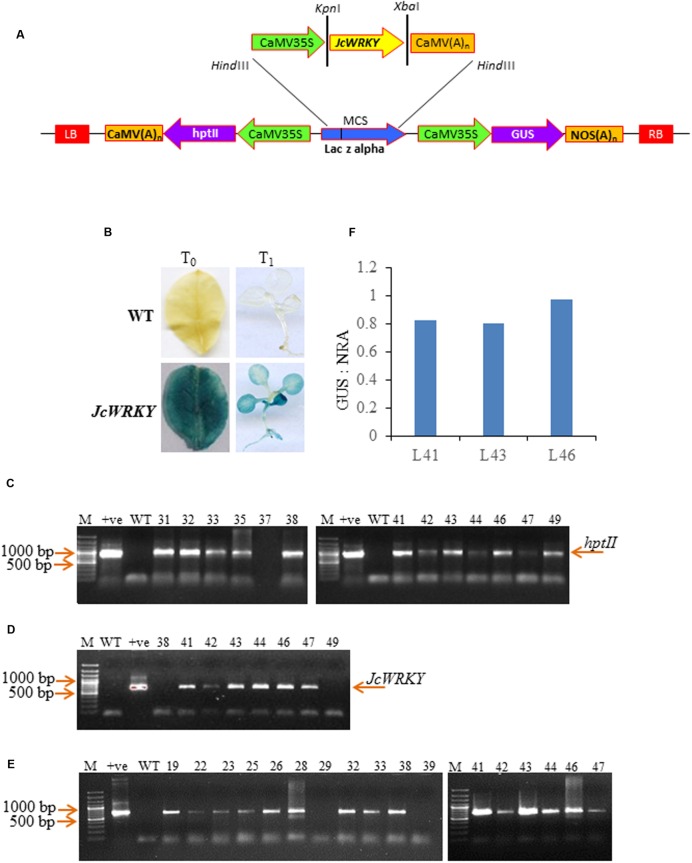
**Characterization of *JcWRKY* transgenic tobacco plants. (A)** Schematic representation of the pCAMBIA1301 – 35S: *JcWRKY* construct used for *Nicotiana tabacum* transformation. **(B)** GUS assay of T_0_ leaf and T_1_ seedlings showing positive expression in the transgenic lines and negative expression in WT plants. PCR confirmation of transgenic lines (lane number denote transgenic line number) with **(C)** *hptII* (964 bp) and **(D)** *JcWRKY* (693 bp) primers. **(E)** Reverse transcriptase PCR of WT and *JcWRKY* transgenics. Amplification was observed with some transgenics, while no amplification with WT. Actin gene was used as an internal control. M refers to 100 bp plus ladder (MBI Fermentas). **(F)** GUS: NRA ratio for determination of copy number of transgene in tobacco plants.

For tobacco transformation, the cDNA of *JcWRKY* (Agarwal et al., 2014, NCBI acc no KC191643), was PCR amplified using JcWRKYTF and JcWRKYTR primers (Supplementary Table S1) containing *Kpn*I and *Xba*I restriction sites, respectively, and the digested fragment was cloned in *Kpn*I/*Xba*I sites of pRT101 vector (Topfer et al., 1987). Thereafter, the entire expression cassette containing 35S: *JcWRKY*: Poly A was cloned in the pCAMBIA 1301 at the *Hind*III site and mobilized into the *Agrobacterium* strain LBA4404. The *Agrobacterium* cells, harboring binary plasmid (**Figure 1A**), were used to transform *Nicotiana tabacum* L. cv. Petit Havana leaf disks according to Horsch et al. (1985). The Murashige and Skoog (1962) medium supplemented with 5 μM BAP (6-benzylaminopurine), 1 μM IAA (indole-3-aceticacid), hygromycin (20 mg/l) and cefotaxime (300 mg/l) was used for regeneration. The multiples shoots were separated and subcultured on MS medium supplemented with 2 μM BAP for 4-weeks. The rooting was carried out on MS basal medium and subsequently hardened to be transferred in green house.

### Histochemical GUS Staining

The Glucuronidase gene (GUS) activity was studied in leaf tissue at T_0_ and T_1_ stage with β-glucuronidase reporter gene staining kit using manufacturer’s protocol (Sigma, USA).

### Confirmation of Gene Integration and Transcript Expression in Tobacco Transgenics

Genomic DNA was isolated from different T_0_ lines by CTAB buffer (Doyle and Doyle, 1987) and used for confirming transgene integration by PCR with hygromycin phosphotransferase (*hptII*), GUS and gene-specific primers (Supplementary Table S1).

Total RNA from WT and transgenics was extracted using Tris-SDS buffer (Agarwal et al., 2015) and quantified with Epoch spectrophotometer (Biotek, India). DNase I (MBI Fermentas) treatment was used for removing genomic DNA contamination. Five microgram of total RNA was used for first strand cDNA synthesis following manufacturer’s protocol (Thermo Scientific). The reverse transcriptase PCR was carried out using 150 ng JcWRKYTF and JcWRKYTR primers or 150 ng actin primers in a 50 μl reaction with the following conditions: 94°C for 5 min, one cycle; 94°C for 1 min, 55°C for 1 min, 72°C for 1 min, 30 cycles; 72°C for 7 min, one cycle. The PCR products were analyzed via agarose gel electrophoresis. The copy number of the transgene was determined by Real-time quantitative PCR using GUS and NRA gene primers as reported earlier in Agarwal et al. (2016a).

### Plant Stress Treatments

To study the germination potential of transgenics (L41, L43, and L46) the T_1_ seeds were subjected to different stress treatments; (a) NaCl: MS medium supplemented with NaCl (0, 100, 150, and 200 mM), (b) salicylic acid (SA): MS medium supplemented with SA (50, 100, 150, and 200 μM). The germination percentage was scored 15 days after seed inoculation.

To study the stress tolerance of *JcWRKY* overexpressing tobacco plants, 15-days-old WT and hygromycin positive T_1_ transgenic seedlings were transferred to plastic cups containing mixture of black soil: red soil: farmyard manure in 1:2:1 ratio, for 30 days. The uniform sized plants were subjected to 200 mM NaCl, 150 μM SA and combined stress of NaCl + SA (200 mM + 150 μM) for a period of 15 days. Since, tobacco is a low level SA plant, therefore low concentration of SA (0.1–0.5 mM) is considered optimal for eliciting stress response (Miura and Tada, 2014). In the present study, 150 μM SA treatment was provided. Thereafter, membrane stability index (MSI), electrolyte leakage (EL) and ion content (ICP), and biochemical parameters [total soluble sugar (TSS), super oxide ($O_{2}^{•-}$), hydrogen peroxide (H_2_O_2_) quantification, superoxide dismutase (SOD) and catalase (CAT)] were recorded.

### Extraction and Quantification of Salicylic Acid from Tobacco Leaves

The plant growth regulators (PGRs) were isolated according to Pan et al. (2010) from tobacco leaf tissue exposed to different treatments and from their corresponding control seedlings. The plant tissue was ground with liquid nitrogen and 50 mg of powdered tissue was transferred into 2 ml Eppendorf tube. The samples for SA determination were prepared in triplicates. DHB (2, 5- dihydroxybenzoic acid) was used as internal standards (1 μg/sample) for SA (Agarwal et al., 2016b). SA was extracted with 500 μl extraction buffer containing 2-propanol, concentrated HCl and water (2:0.002:1) at 4°C for 30 min (Pan et al., 2010). The HPLC analysis was carried out as in Agarwal et al. (2016b).

### Analysis of Physiological and Biochemical Parameters of Transgenics in Response to Stress

The MSI, EL, TSS, $O_{2}^{•-}$, and H_2_O_2_ quantification, antioxidative enzymatic analysis of CAT, SOD and ion content were measured. The MSI, EL, TSS were carried out as per Shukla et al. (2012).

#### Quantification of $O_{2}^{•-}$ and H_2_O_2_ Content

$O_{2}^{•-}$ content was determined according to Shukla et al. (2012). Leaf tissue was extracted in 10 ml of 65 mM potassium phosphate buffer (pH 7.8) and centrifuged at 5,000 × *g* for 10 min. The reaction mixture containing 0.9 ml of 65 mM phosphate buffer (pH 7.8), 0.1 ml of 10 mM hydroxylamine hydrochloride, and 1 ml of the extract was incubated at 25°C for 20 min. Then 17 mM sulfanilamide and 7 mM α-naphthylamine were added, further, incubated at 25°C for 20 min and the absorbance was read at 530 nm. A standard curve (10–200 nmol) was prepared with NaNO_2_ to calculate the production rate of $O_{2}^{•-}$.

The H_2_O_2_ content in leaf samples was measured as described by Shukla et al. (2012). Leaf tissue was extracted with cold acetone to determine the H_2_O_2_ levels. 2 ml of the extract was mixed with 0.5 ml of 0.1% titanium dioxide in 20% (v:v) H_2_SO_4_ and the mixture was then centrifuged at 6,000 × *g* for 15 min. The intensity of yellow color of the supernatant was measured at 415 nm and the concentration of H_2_O_2_ was calculated against the standard curve.

#### Determination of Enzyme Activities

Leaf tissue of 1–2 gm from WT and transgenics was homogenized in 15 ml of 50 mM phosphate buffer, pH 7.0, containing 1% polyvinylpyrrolidone. The homogenate was centrifuged at 15, 000 × *g* for 10 min and the supernatant obtained was used as enzyme extract.

#### Superoxide Dismutase (EC1.15.1.1)

Superoxide dismutase activity was measured as reported in Dhindsa et al. (1981) with minor modification, based on the potential to inhibit the photoreduction of nitro blue tetrazolium (NBT). The 3 ml reaction mixture contained 100 mM phosphate buffer, pH 7.5, 200 mM methionine, 2.25 mM NBT, 60 μM riboflavin, 3.0 mM EDTA, and 50 μl enzyme extract. The reaction was carried out at room temperature for 15 min under bright light (2 × 15 W fluorescent lamps), and absorbance was recorded at 560 nm. The log A_560_ was plotted as a function of the volume of enzyme extract (Giannopolitis and Ries, 1977), and the volume of enzyme extract corresponding to 50% inhibition of the reaction was considered as one enzyme unit (Beauchamp and Fridovich, 1971).

#### Catalase (EC1.11.1.6)

Catalase assay was carried out by measuring the initial rate of disappearance of H_2_O_2_ as reported by Dhindsa et al. (1981). The 3 ml reaction mixture contained 100 mM phosphate buffer, pH 7.5, 75 mM H_2_O_2_, and 50 μl enzyme extract. The decrease in H_2_O_2_ was observed as a decline in A_240_ using Epoch spectrophotometer (Biotek, India) and activity was expressed in units (1 unit defines the conversion of one mole of H_2_O_2_/minute).

#### Analysis of Ion Content

The ion content was studied using inductively coupled plasma optical emission spectrometer (Optima 2000DV, Perkin Elmer, Germany) as in Shukla et al. (2012).

### Real-Time PCR Analysis of Downstream Genes

The quantitative expression of downstream genes (**Table 1**) was studied by real-time PCR. The cDNA was synthesized from the stress-treated and control WT and T_1_ transgenic plants as previously mentioned. The cDNA was used as a template for qPCR analysis. Actin (NtActin F and NtActin R primers) was used as an internal control gene. Real-time PCR was performed with SYBR green jumpstart Taq ready mix (Sigma-Aldrich) with following PCR condition: 94°C, 2 min for one cycle; 94°C, 60 s, 55°C, 60 s and 72°C, 60 s for 45 cycles. The specificity of PCR amplification was checked by melt curve analysis at the end of the PCR cycles. Each reaction was replicated three times and the relative-fold expression was determined using Livak method (Livak and Schmittgen, 2001).

**Table 1:** List of selected genes studied by real time PCR for transcript regulation in tobacco transgenics.

S. no.	Target gene name/accession number	Primer sequence (5′→3′)	Functions
1	NtSOD/ AF443178	F:TGCAGCTCCACCACCAGAAGCATCATCAGAC R:GGCTCACCACCACCCTCGCGGACA	Antioxidative enzyme
2	NtCAT/HF564632	F:AGGTACCGCTCATTCACACC R:AAGCAAGCTTTTGACCCAGA	Antioxidative enzyme
3	NtICS/EU257505	F:CAGTTCTGTTTGCAACCTCC R:GAGTAGGGTGAATGGACGAC	SA biosynthetic gene

### Statistical Analyses

Each experiment was repeated thrice and the mean values and standard deviations were calculated. Analysis of variance was calculated using Fishers least significant difference (LSD) by Infostat software at *P* ≤ 0.05 to determine the significance of difference between the means of control and different stress treatments. Mean values of treatments that were significantly different from each other were indicated by different alphabets. Correlation coefficient between physio-biochemical parameters was determined using PAST (Palaeontological STatistics, ver. 1.89) as reported by Sarkar et al. (2016).

## Results

### Overexpression of *JcWRKY* Confer Enhanced Stress Tolerance

#### Analysis of T_0_ Transgenic Lines

To study the role of JcWRKY toward stress tolerance, transgenic tobacco overexpressing *JcWRKY* were generated. Among 58 hygromycin resistant plants, 35 showed histochemical GUS staining (**Figure 1B**). Some plants showed proper blue color in leaves while others showed scattered blue spots. The GUS positive plants showed the presence of *JcWRKY* and hygromycin genes (**Figures 1C,D**). Transgenic lines showed expression of the *JcWRKY* gene by reverse transcriptase PCR, but the corresponding band was not observed in WT plants (**Figure 1E**). The presence of a single transcript indicated that transcription initiation and termination of *JcWRKY* mRNA occurred as expected. The transgenic plants (T_0_) showed no morphological difference in the vegetative and floral tissues, as compared to WT plants. The transgenic lines (L41, L43, L46) with high transcript expression of the gene were selected for functional validation toward stress tolerance by physio-biochemical and molecular analyses.

Seeds from T_0_ plants were analyzed for Mendelian principle of segregation. Transgenic lines followed Mendelian segregation ratio of 3:1 Hyg^r^/Hyg^s^ (**Table 2**). The real-time PCR showed the presence of single copy of the transgene in transgenics L41, L43, and L46 with GUS:NRA ratio of 0.826, 0.802, 0.975, respectively (**Figure 1F**). The ratio 0.5–1.0 indicate the presence of the single transgene.

**Table 2:** Segregation ratio of transgenics.

Transgenic lines	No. of seeds inoculated	No. of seeds germinated	Ratio HYG^r^: HYG^s^
L41	344	109	3.155
L43	342	111	3.081
L46	310	99	3.131

**FIGURE 2:**
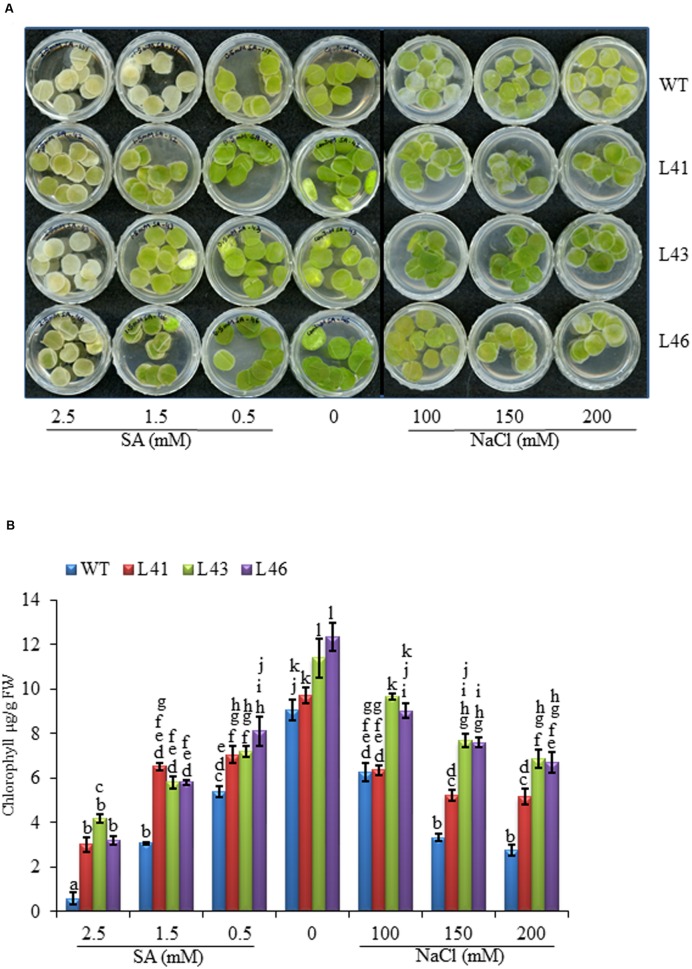
**(A)** Leaf disk assay of WT and T_0_ transgenic lines (L41, L43, and L46) at different concentrations of NaCl (0, 100, 150, 200 mM) and SA (0.5, 1.5, 2.5 mM). **(B)** Chlorophyll analysis at different SA and NaCl concentrations. Values are represented as mean ± SD (*n* = 3) and marked with different alphabets to indicate significant difference at *P* ≤ 0.05 probability.

Leaf disk of uniform size from WT, L41, L43, and L46 were inoculated in the solution of NaCl (100, 200, and 300 mM), SA (0.5, 1.5, 2.5 mM) and water (control). After 7 days, WT leaf disks started turning pale in the presence of NaCl and SA, in contrast, leaf disk from transgenics remained green (**Figure 2A**). The chlorophyll content in transgenic leaf disks were significantly higher in all the concentrations of NaCl and SA as compared to WT. The 2.5 mM SA showed highly reduced chlorophyll content both in transgenics and WT, although the reduction was more pronounced with WT (**Figure 2B**).

#### Analysis of T_1_ Transgenic Lines

The T_1_ transgenic progeny was studied to establish the stress tolerance potential of tobacco transgenics overexpressing *JcWRKY* gene.

##### Seed germination

**FIGURE 3:**
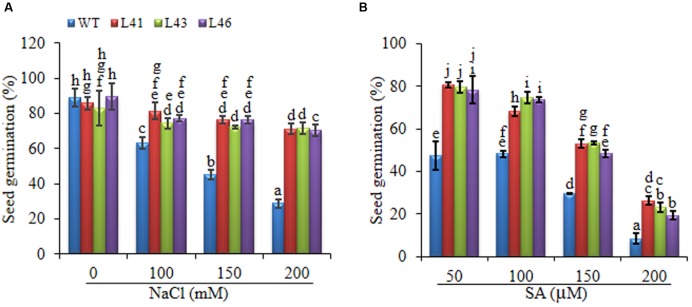
**Percentage seed germination at on different concentrations of (A) NaCl (0, 100, 150, 200 mM) and (B) SA (50, 100, 150, 200 μM).** Values are represented as mean ± SD (*n* = 3) and marked with different alphabets to indicate significant difference at *P* ≤ 0.05 probability.

To study the effect of salt stress on germination, the WT and T_1_ (L41, L43, and L46) seeds were germinated on MS medium supplemented with NaCl (0, 50, 100, 200 mM). At 0 mM concentration the germination of WT and transgenic lines was similar, however, with salt stress, the transgenic seeds showed earlier and higher percent germination as compared to WT seeds. The percent of seed germination reduced with increasing concentration of NaCl in both WT and transgenics (**Figure 3A**), at 150 mM NaCl the WT showed 45% germination whereas, the transgenics showed 72–76% germination, further at 200 mM NaCl, germination percentage was reduced to 29% and 70–71% in WT and transgenics, respectively. On 300 mM NaCl, the transgenics showed very weak germination and the WT failed to germinate till 15-day of seed inoculation.

With SA treatment, the transgenics showed better germination as compared to WT. The germination in WT was 47% at 50 μM SA which decreased to 8% on 200 μM SA, whereas, the transgenics showed 78–80% and 19–26% on 50 and 200 μM SA, respectively. On 250 μM SA both WT and transgenics showed no germination (**Figure 3B**).

#### Physiological and Biochemical Response of *JcWRKY* Transgenics in Response to Stress

**FIGURE 4:**
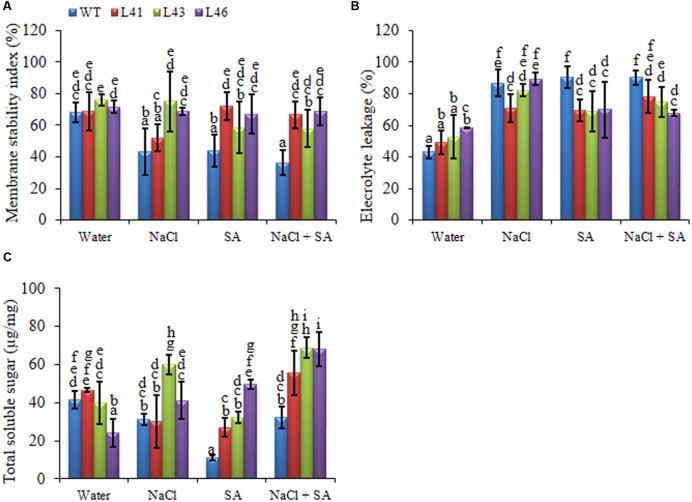
**Biochemical changes in WT and *JcWRKY* transgenic plants grown in the presence of 0, 200 mM NaCl, 150 μM SA, and combined NaCl + SA stress. (A)** Membrane stability index, **(B)** Electrolyte leakage and **(C)** Total soluble sugar content. Values are represented as mean ± SD (*n* = 3) and marked with different alphabets to indicate significant difference at *P* ≤ 0.05 probability.

The 15-days-old WT and T_1_ transgenic seedlings were transferred to plastic cups containing soil for 30 days. The uniform sized plants were subjected to 0 mM, 200 mM NaCl, 150 μM SA and a combined stress of 200 mM NaCl + 150 μM SA for a period of 15 days. Transgenic lines exhibited better osmotic adjustment and physiological status as compared to WT plants. The MSI of transgenics was significantly higher than WT, indicating better membrane stability, transgenics exhibited an average of 1.5 -fold higher MSI with NaCl and SA stress, whereas the combined treatment showed to 1.7 -fold higher MSI (**Figure 4A**). Maximum reduction of EL was observed in L46 (24.7%) on combined stress treatment (**Figure 4B**). Accumulation of TSS was higher in trangenics to facilitate osmoregulation, the average accumulation of 1.4, 3.2, 2.0-fold was observed with NaCl, SA and combined stress, respectively (**Figure 4C**).

**FIGURE 5:**
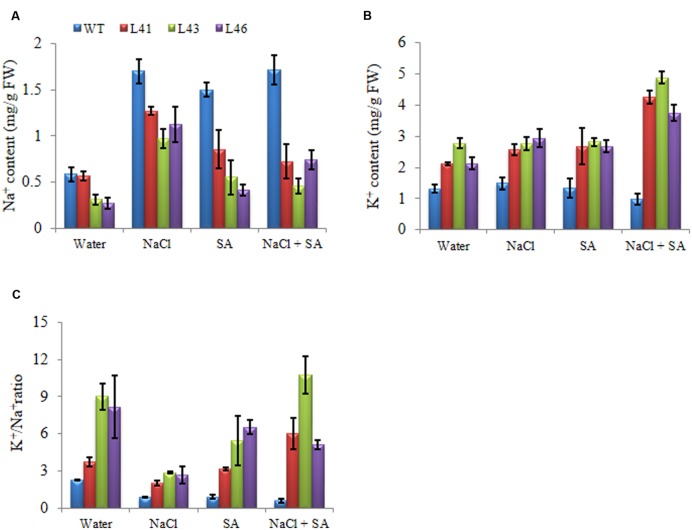
**Analysis of ion content in leaves of WT and *JcWRKY* transgenic tobacco plants with NaCl, SA and combinatorial stress. (A)** Na^+^ content, **(B)** K^+^ content, **(C)** K^+^/Na^+^. Values are represented as mean ± SD (*n* = 3).

Accumulation of different ions was analyzed in WT and transgenics in response to different stresses. At 0 mM NaCl, Na^+^ content was less in WT as compared to transgenics. In the presence of NaCl or SA, all plants showed enhanced accumulation of Na^+^ ions, however the transgenics accumulated significantly lower Na^+^ content than WT (**Figure 5A**). The K^+^ content was higher in transgenic lines when exposed NaCl or SA and was further increased by combinatorial stress (**Figure 5B**) and thus helped to maintain higher K^+^/Na^+^ (**Figure 5C**).

**FIGURE 6:**
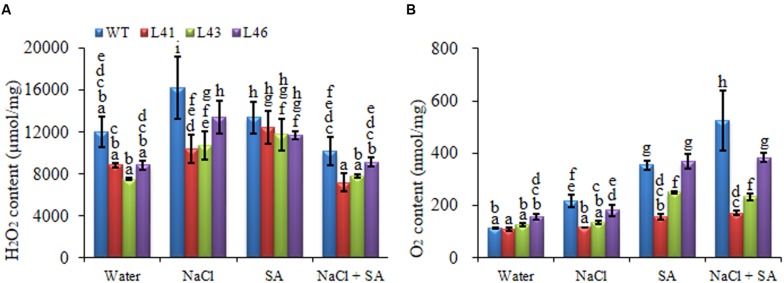
**Quantification of (A) H_2_O_2_ and (B) $O_{2}^{•-}$ in leaves of WT and transgenic lines in the presence of 0, 200 mM NaCl, 150 μM SA, and combined NaCl + SA stress.** Values are represented as mean ± SD (*n* = 3) and marked with different alphabets to indicate significant difference at *P* ≤ 0.05 probability.

**FIGURE 7:**
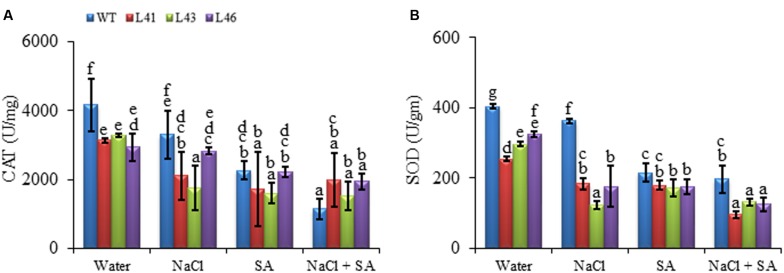
**Activity of (A) CAT and (B) SOD in WT and *JcWRKY* transgenic lines (L41, L43, and L46) at different NaCl, SA and combined stress treatments.** Values are represented as mean ± SD (*n* = 3) and marked with different alphabets to indicate significant difference at *P* ≤ 0.05 probability.

During abiotic stress conditions, ROS get accumulated in plant tissues leading to oxidative damage. The $O_{2}^{•-}$ and H_2_O_2_ content of WT was significantly higher with all the treatments as compared to the transgenics. The NaCl treatment resulted in higher H_2_O_2_ content in both WT (16208 nmol/mg FW) and transgenics (L41, L43, L46; 10376, 10752, 13457 nmol/mg FW, respectively), however, the content was significantly lower in the transgenics (**Figure 6A**). Interestingly, the combined stress treatment resulted in highest accumulation of $O_{2}^{•-}$ in WT (526.08 nmol/mg FW), however, the transgenics showed significantly reduced almost similar accumulation (ranging from 159 to 353 nmol/mg FW) with SA only and combined stress (**Figure 6B**). The ROS-scavenging enzymes, CAT and SOD, activity declined in both WT and transgenics on exposure to stress treatments (**Figures 7A,B**). The SOD content of WT showed a decline of 10, 46 and 51%, whereas, the transgenics showed an average decline of 44, 39, and 60% with NaCl, SA and combined stress, respectively. The WT showed minimum CAT activity of 1142 U/mg FW on combined stress treatments, indicating a decrease of 73% activity compared to control conditions, whereas, the transgenics showed significantly higher activity with combined stress.

### SA Phytohormone Analysis in *JcWRKY* Transgenics

**FIGURE 8:**
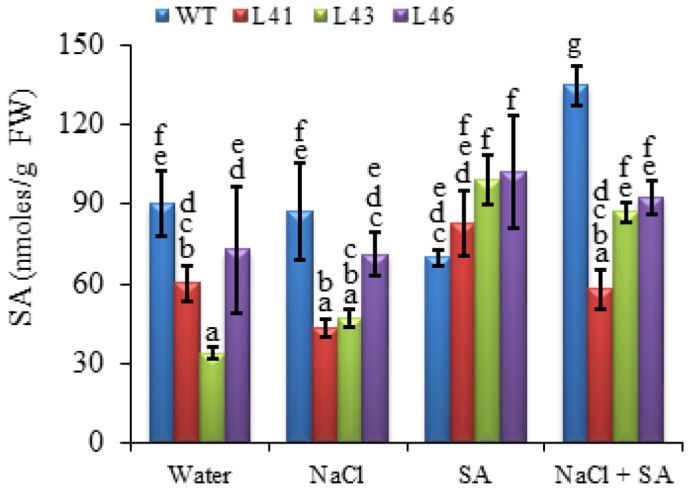
**The SA concentration in WT and *JcWRKY* transgenic lines (L41, L43, and L46) at different NaCl, SA and combined stress treatments.** Values are represented as means ± SD (*n* = 3) and marked with different alphabets to indicate significant difference at *P* ≤ 0.05 probability.

The SA phytohormone was quantified in WT and transgenics, and it was observed that the transgenics show increased SA content (81–99 nmoles/mg FW) as compared to WT (70 nmoles/mg FW)with SA treatment only, whereas with other treatments WT shows higher SA content. The WT showed maximum SA accumulation (133.9 nmoles/mg FW) on combined stress treatment, whereas, the transgenics showed accumulation in the range of 58–90 nmoles/mg FW (**Figure 8**).

**FIGURE 9:**
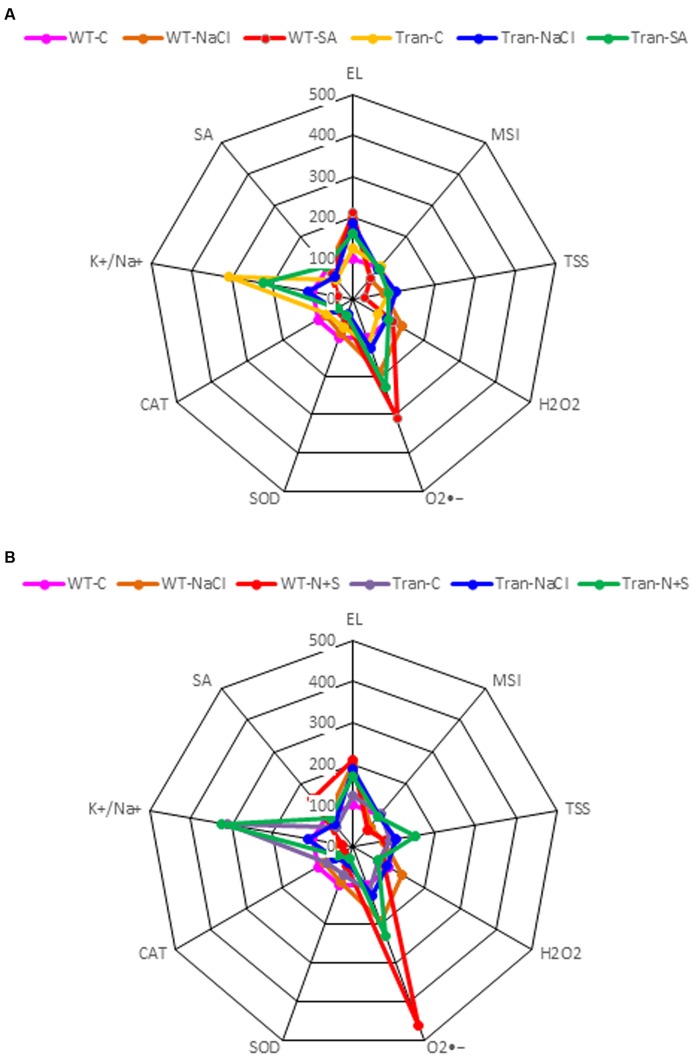
**The radar diagram built by comparing all the physio-biochemical parameters between WT and transgenic plants (average of three transgenic lines) (A) NaCl, SA and control, (B) NaCl, combinatorial (NaCl + SA) stress and control**.

The radar diagram was built by comparing all the physio-biochemical parameters (EL, MSI, TSS, $O_{2}^{•-}$, H_2_O_2_, SOD, CAT, K^+^/Na^+^ ratio) between WT and transgenic plants under control, salinity and SA (**Figure 9A**) treatment and also with control, salinity and combinatorial treatment (**Figure 9B**). The H_2_O_2_ content was higher in WT with NaCl treatment as compared to transgenics, and was further reduced in transgenics on combinatorial stress. The highest accumulation of $O_{2}^{•-}$ is observed in WT with SA and combinatorial stress, also the WT show a low K^+^/Na^+^ ratio as compared to transgenics.

### Differential Regulation of Downstream Genes in *JcWRKY* Transgenics

**FIGURE 10:**
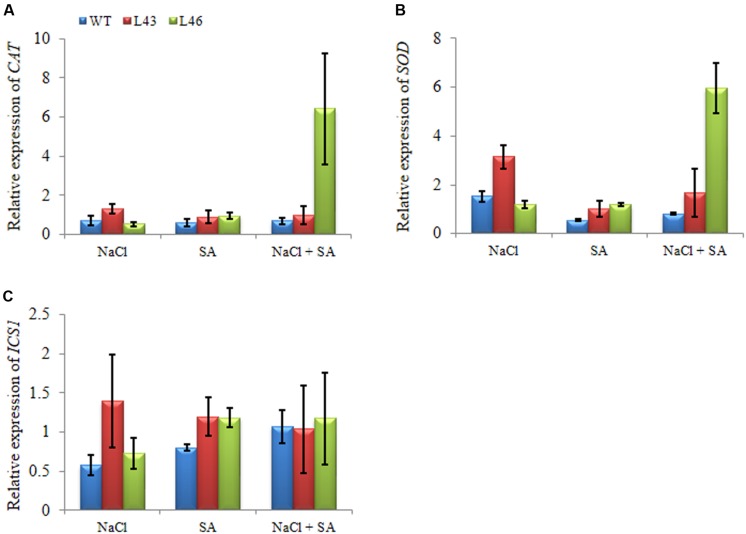
**Relative-fold expression of downstream genes in *JcWRKY* transgenics under different stress by real-time PCR; (A) *CAT*, (B) *SOD*, and (C) *ICS1.*** Values are represented as mean ± SE (*n* = 3).

To study putative molecular mechanisms of *JcWRKY* overexpression for enhanced stress tolerance, expression of *CAT* and *SOD* genes and SA biosynthetic gene, isochorismate synthase 1 (*ICS1*) were studied. Under all three treatments, the *CAT* gene showed higher accumulation of transcript in transgenic lines (L43, L46) than WT. During salt treatment, the slight upregulation of CAT transcript was observed in transgenics, whereas with the combined stress L46 showed an expression of 6.43 fold (**Figure 10A**). Similar trend of relative expression was observed with *SOD* transcript. With combined stress L46 showed maximum *SOD* transcript expression of 6 fold (**Figure 10B**). Thus, L46 line showed maximum accumulation of both *CAT* and *SOD* transcript during combined stress.

The *ICS1* transcript showed higher accumulation in transgenic lines as compared to WT under all the treatments, however, with combined stress similar transcript was observed in WT and transgenics (1.0–1.17-fold,**Figure 10C**).

### Evaluation of Physio-Biochemical Parameters under Stress Conditions

The correlation coefficient between different physio-biochemical parameters in WT and transgenics exposed to salinity (**Table 3**), SA (**Table 4**) and combinatorial stress (**Table 5**) was analyzed. With salinity stress, the EL showed positive correlation with $O_{2}^{•-}$ (0.56) and MSI was positively correlated with TSS (0.57), K^+^/Na^+^ ratio (0.71). TSS also showed positive correlation with K^+^/Na^+^ ratio (0.56). With SA treatment, the antioxidative enzymes, SOD and CAT showed positive correlation with EL (0.64) and H_2_O_2_ (0.577), respectively. The combinatorial stress resulted in K^+^/Na^+^ ratio showing positive correlation with TSS and negative correlation with $O_{2}^{•-}$, H_2_O_2_, and SOD. The CAT and SOD showed negative correlation with combined stress, whereas were positively regulated on salinity stress.

**Table 3:** Correlation coefficient(r) between biochemical parameters of transgenic and WT plants exposed to 200 mM NaCl stress.

	EL	MSI	TSS	$O_{2}^{•-}$	H_2_O_2_	SOD	CAT	K^+^/Na^+^
EL	1.00							
MSI	0.20208	1.00						
TSS	-0.01803	0.57156^∗^	1.00					
$O_{2}^{•-}$	0.56506^∗^	-0.40219	-0.21321	1.00				
H_2_O_2_	0.1742	-0.33053	-0.04225	0.75379^∗∗^	1.00			
SOD	0.16704	-0.63757^∗∗^	-0.51527	0.76228^∗∗^	0.66948^∗∗^	1.00		
CAT	0.43933	-0.55765^∗^	-0.27592	0.73148^∗∗^	0.45928	0.65534^∗^	1.00	
K^+^/Na^+^	-0.0331	0 0.71128^∗∗^	0.5688^∗^	-0.5634^∗^	-0.52176	-0.9266^∗∗^	-0.53739	1.00

*For each parameter, average values of three WT and JcWRKY transgenic tobacco. Where, EL, electrolyte leakage; MSI, membrane stability index; TSS, total soluble sugar; $O_{2}^{•-}$, Superoxide radical content; H_2_O_2_, hydrogen peroxide content; SOD, superoxide dismutase; CAT, catalase; K^+^/Na^+^, ion content ratio. Where, ^∗^ and ^∗∗^ indicates significant and highly significant correlation at ^∗^P ≤ 0.05 and ^∗∗^P ≤ 0.01, respectively.*

**Table 4:** Correlation coefficient(r) between biochemical parameters of transgenic and WT plants exposed to 150 μM SA stress.

	EL	MSI	TSS	$O_{2}^{•-}$	H_2_O_2_	SOD	CAT	K^+^/Na^+^
EL	1.00							
MSI	-0.19704	1.00						
TSS	-0.45751	0.53366	1.00					
$O_{2}^{•-}$	0.36511	-0.36581	0.13468	1.00				
H_2_O_2_	0.33085	0.029922	-0.34809	0.30423	1.00			
SOD	0.64186^∗^	-0.71374^∗∗^	-0.54695	0.27308	-0.01808	1.00		
CAT	-0.02826	-0.29983	-0.09929	0.51824	0.5777^∗^	0.13499	1.00	
K^+/^Na^+^	-0.65956^∗∗^	0.29922	0.88079^∗∗^	0.07291	-0.54204	-0.53321	-0.09459	1.00

*For each parameter, average values of three WT and JcWRKY transgenic tobacco. Where, EL, electrolyte leakage; MSI, membrane stability index; TSS, total soluble sugar; $O_{2}^{•-}$, superoxide radical content; H_2_O_2_, hydrogen peroxide content; SOD, superoxide dismutase; CAT, catalase; K^+^/Na^+^, ion content ratio. Where, ^∗^ and ^∗∗^ indicates significant and highly significant correlation at ^∗^P ≤ 0.05 and ^∗∗^P ≤ 0.01 respectively.*

**Table 5:** Correlation coefficient(r) between biochemical parameters of transgenic and WT plants exposed to combinatorial (NaCl + SA) stress.

	EL	MSI	TSS	$O_{2}^{•-}$	H_2_O_2_	SOD	CAT	K^+^/Na^+^
EL	1.00							
MSI	-0.62176^∗^	1.00						
TSS	-0.7618^∗∗^	0.6708^∗∗^	1.00					
$O_{2}^{•-}$	0.35273	-0.6525^∗^	-0.44079	1.00				
H_2_O_2_	0.20019	-0.30512	-0.22798	0.6918^∗∗^	1.00			
SOD	0.59275^∗^	-0.69003^∗∗^	-0.47687	0.65395^∗^	0.80425^∗∗^	1.00		
CAT	-0.11286	0.43041	0.27464	-0.35295	-0.52011	-0.57388^∗^	1.00	
K^+/^Na^+^	-0.40287	0.49106	0.57413^∗^	-0.75151^∗∗^	-0.59457^∗^	-0.56908^∗^	0.33074	1.00

*For each parameter, average values of three WT and JcWRKY transgenic tobacco. Where, EL, electrolyte leakage; MSI, membrane stability index; TSS, total soluble sugar; $O_{2}^{•-}$, superoxide radical content; H_2_O_2_, hydrogen peroxide content; SOD, superoxide dismutase; CAT, catalase; K^+^/Na^+^, ion content ratio. Where, ^∗^ and ^∗∗^ indicates significant and highly significant correlation at ^∗^P ≤ 0.05 and ^∗∗^P ≤ 0.01 respectively.*

## Discussion

Transcriptional control is a major mechanism regulating the cellular processes during stress signaling. Stress sensing triggers large scale transcriptional programing for minimizing the deleterious effect of stress (Nakashima et al., 2014). Since, the plants are exposed to multiple stress simultaneously, therefore transcriptional control facilitates to regulate the complex patterns of gene expression during multiple stresses. The research on stress signaling is mainly focused on individual stress treatments, however, understanding of combinatorial stress is important for developing crop resilence toward environmental conditions (Kissoudis et al., 2014). The phytohormones, signaling molecules, TFs, ROS, ion fluxes are the major components of the regulatory network that crosstalk and converge to provide protective responses of plants against different stresses. The WRKY TFs interact with other TFs, or with WRKY themselves and also directly regulate some functional genes to impart stress tolerance (Agarwal et al., 2011). Salinity stress generates both hyperosmotic and hyperionic stress, the osmotic stress or physiological dehydration being imposed at an early phase and ionic stress at a later stage of plant growth (Shavrukov, 2013). The role of SA phytohormone is well-characterized toward regulating the plant defense responses, however, it also ameliorates toxicity induced by salinity, but the downstream mechanism involved in imparting salinity tolerance remains unclear (Wani et al., 2016). The SA functions as a universal signaling component for both abiotic and biotic stresses. The effect of exogenous SA on plant responses/processes is greatly dependent on concentration and plant species. The application of the SA in appropriate concentration, enhances stress tolerance (Miura and Tada, 2014). Usually low concentrations of SA might enhance the antioxidant capacity, however, high concentrations lead to cell death or susceptibility to abiotic stresses (Hara et al., 2012). Kang et al. (2014) reported that SA participates in abiotic stress-induced signaling by improving the antioxidant capacity of the plant.

In this study, *JcWRKY* transgenics showed enhanced tolerance toward salinity, which, further improved on application of low SA concentration. The detailed analysis of transgenic plants reveals the possibility that JcWRKY regulates ROS homeostasis via SA signaling for combating stress. The *JcWRKY* transgenics showed higher chlorophyll content and seed germination under salinity. Similarly, earlier reports revealed the involvement of WRKY in stress tolerance in different plant system. A comprehensive microarray analysis of the *Arabidopsis* root transcriptome showed regulation of different WRKY during salinity stress (Jiang and Deyholos, 2006). The AtWRKY25 and AtWRKY33 form a transcriptional network independent of SOS signaling and confer salt tolerance to transgenics (Jiang and Deyholos, 2009). The *GmWRKY54* transgenic *Arabidopsis* showed salt/drought tolerance, possibly through the regulation of TF like DREB2A and STZ/Zat10 (Zhou et al., 2008). The *Arabidopsis* WRKY8 functions antagonistically with its interacting partner VQ9 for appropriate maintenance of WRKY8 TF signaling during salt stress (Hu et al., 2013). The overexpression of WRKY TFs is also found associated with improved systemic acquired resistance and SA signaling (Li et al., 2004; Vallad and Goodman, 2004; Agarwal et al., 2011). We observed that the SA assisted toward better control of salt stress and did not result in any detrimental effect on the transgenics, on the contrary, it facilitated better growth with improved TSS, MSI and reduced EL. Similarly, the *TaWRKY2* and *TaWRKY19* transgenics showed higher soluble sugar and reduced EL (Niu et al., 2012). The *JcWRKY* transgenics showed low levels of $O_{2}^{•-}$ and H_2_O_2_, similarly tobacco transgenics overexpressing *TaWRKY10* gene showed reduced levels of $O_{2}^{•-}$ and H_2_O_2_ on exposure to salinity and drought stresses (Wang et al., 2013). The accumulation of $O_{2}^{•-}$ showed an additive effect in WT on combined stress treatment as compared to water or NaCl treatment, whereas, in the transgenics the additive accumulation of $O_{2}^{•-}$ was not observed, indicating that SA participates more efficiently in *JcWRKY* transgenics for regulating the ROS homeostasis through SA mediated signaling. ROS homeostasis is maintained by a balance of ROS generation and its scavenging (Apel and Hirt, 2004). ROS represent a common signal and significant point of convergence between stress signaling pathways (Fujita et al., 2006). The SA plays an equivocal role in promoting ROS accumulation (prooxidant) and also toward ROS scavenging (antioxidant) during different stress conditions (Miura and Tada, 2014). SA modulates ROS homeostasis by regulating plant antioxidant machinery and also via transcriptional regulation of genes.

The MAPK (MAP kinase) cascade also participates in SA or ROS signaling to regulate downstream genes (Rodriguez et al., 2010), furthermore certain SA or ROS activated MAKs also regulate abiotic stress response (Asai et al., 2002). The WRKY family of TFs that bind to the W-box (TTGAC(C/T) participate in SA-mediated transcriptional programming (Pandey and Somssich, 2009). The WRKY25 and WRKY33 TFs may act downstream of the MPK4-mediated signaling and contribute toward SA-dependent disease resistance response (Andreasson et al., 2005). Shen et al. (2012), reported phosphorylation of OsWRKY30 by MAPK cascade for enhanced drought tolerance in rice, WRKY TFs act downstream of various MAPKs (Asai et al., 2002; Liu et al., 2004) in regulating plant defense gene activation. We have earlier reported the presence of seven potential phosphorylation sites in JcWRKY, the binding of the SA-inducible JcWRKY TF to the W-box of PR-1 and iso1 promoter (Agarwal et al., 2014).

The *JcWRKY* transgenics showed low Na^+^ concentration in tissues, thereby maintained high K^+^/Na^+^, an important feature required for growth and tolerance during hyperionic stress. With salinity stress, the entry of Na^+^ ions lowers the cytosolic K^+^ content required for various cellular metabolic processes (Shabala, 2009). Jayakannan et al. (2013) report that SA improves salinity tolerance in *Arabidopsis*, pretreating roots with SA decreased K^+^ loss through guard cell outward-rectifying K^+^ channel (GORK) or by ROS-activated non-selective cation channels (NSCCs). The SA pretreatment is also reported to up-regulate the plasma membrane H^+^-ATPase activity (Liu et al., 2009). Thus, cross talk of SA and ROS also participates in ion transport processes during salinity stress.

Plants contain a complex antioxidative defense system, antioxidative enzymes are key components of ROS scavenging system. SOD is a major scavenger of $O_{2}^{•-}$ it catalyzes the formation of H_2_O_2_ and $O_{2}^{•-}$. The H_2_O_2_ thus produced is scavenged by CAT. Upregulation of *CAT* and *SOD* genes in *JcWRKY* transgenics show their involvement in ROS scavenging. The W-box sequence of WRKY is also reported in the promoters of a number of superoxide inducible genes (Scarpeci et al., 2008). The SOD activity of *JcWRKY* transgenics is low as compared to WT, which could be due to the reduced $O_{2}^{•-}$ accumulation in the transgenics. The CAT activity is reduced in both WT and transgenics on NaCl/SA application, however, with combined stress the CAT activity of transgenics improves, but its activity gets further reduced in WT, indicating the protective role of SA in the transgenics. Interestingly, on combinatorial stress the CAT and SOD showed negative correlation, whereas were positively regulated on salinity stress (**Tables 3** and **5**). The relationship between SA and H_2_O_2_ during stress condition is defined by a “self amplifying feedback loop” concept, wherein, H_2_O_2_ induces SA accumulation, and SA results in enhanced H_2_O_2_ concentration (Shirasu et al., 1997). The enhanced H_2_O_2_ concentration by SA is due to inhibition of CAT (Durner and Klessig, 1996).

The WRKY28 TF positively regulates the expression of *ICS1* involved in SA biosynthesis pathway (van Verk et al., 2011). The transcript expression of the *ICS1* was upregulated in the *JcWRKY* transgenics, however, interestingly, transgenics maintained the SA phytohormone level during stress conditions and the SA content of the transgenics was higher than WT only on exogenous SA treatment. It can be speculated that the SA and ROS self amplifying feedback loop, facilitated the maintenance of SA and ROS during stress. The OsWRKY13 TF activates both SA synthesis-related genes and SA-responsive genes, suggesting that OsWRKY13 activates both upstream and downstream components of SA (Qiu et al., 2007).

## Conclusion

The understanding and deployment of different strategies toward developing resilient crops that maintain their vigor and productivity with diverse environmental conditions is of prime importance toward generating/facilitating economic sustainable agriculture. Developing resistance to combinatorial stress is challenging and encouraging via involvement of TFs through fine tuning of downstream targets to achieve the desired response. Approaches that result in ROS homeostasis, provide a potential strategy for engineering crop stress tolerance against multiple or combinatorial stresses. In the present study, we highlight the regulatory role of JcWRKY toward improved salinity and also oxidative stress. The tolerance of transgenics, as evident by different physiological and biochemical parameters including low Na^+^/ K^+^ ratio under salinity stress, may be due to improved SA signaling coupled with ROS homeostasis. The transgenics show better tolerance potential to individual as well as combined stresses, indicating improved SA-mediated transcriptional maintenance of ROS homeostasis. Thus, *JcWRKY* can be deployed to engineer stress tolerant transgenic crops for sustained growth and productivity under unfavorable conditions.

## Author Contributions

PA conceived the study, performed gene cloning, data analysis. PA and PKA designed and coordinated the experiments and did manuscript writing. MD carried out tobacco transformation, KS involved in biochemical experiments, PJ involved in real-time expression of transgenics. All authors have read and approved the final manuscript.

## Conflict of Interest Statement

The authors declare that the research was conducted in the absence of any commercial or financial relationships that could be construed as a potential conflict of interest.
