# Supplementary material for: Ectopic Expression of JcWRKY Transcription Factor Confers Salinity Tolerance via Salicylic Acid Signaling
Source: Front Plant Sci. 2016 Oct 17;7:1541. doi: 10.3389/fpls.2016.01541 (PMC5065966; doi:10.3389/fpls.2016.01541)
Supplement: Supplementary file 1 [file Table_1.DOCX]

**Table S1** List of the primers used in this study

| **Primer ID** | | **Primer Sequence (5’-3’)** | |  |  |
| --- | --- | --- | --- | --- | --- |
| JcWRKYTF  JcWRKYTR  hptII F  hptII R  NtActin F  NtActin R  Gus F  Gus R | | CTCGGTACCATGCAGGGGGAATATGAG  TGCTCTAGATTACAATTTTGTAGACTC  GCTCGAGATGGCTGTTACTGTCTTT  TGCTCTAGATTAGTAGGCATTAGGATT  AATGCTGGCACTGATTGCAC  TCCTCTGCGCTGGAACAAG  GATCGCGAAAACTGTGGAAT  TGAGCGTCGCAGAACATTAC | |  |  |
|  | |  | |  | |
